# Time-Domain Numerical Simulation and Experimental Study on Pulsed Eddy Current Inspection of Tubing and Casing

**DOI:** 10.3390/s23031135

**Published:** 2023-01-18

**Authors:** Xingxing Yu, Ying Zhu, Yan Cao, Juan Xiong

**Affiliations:** 1 College of Aviation Electromechanical Equipment Maintenance, Changsha Aeronautical Vocational and Technical College, Changsha 410124, China; 2 Key Laboratory of Nondestructive Testing, Ministry of Education, Nanchang Hangkong University, Nanchang 330063, China; 3 State Key Laboratory of Acoustics, Institute of Acoustics, Chinese Academy of Sciences, Beijing 100190, China

**Keywords:** pulsed eddy current, corrosion, tubing and casing, finite element

## Abstract

Fundamental theory and methods are investigated of inspecting tubing and casing simultaneously using pulsed eddy current testing by numerical simulations and experiments. The distribution and variation of eddy current field are given in the finite element simulation for the inspection of undamaged and corroded casing and tubing combinations, with tubing outer diameter 73.8 mm, wall thickness 5.7 mm, corrosion depth 1.25 mm, 2.5 mm, 3.75 mm, and casing outer diameter 141.5 mm, wall thickness 7.7 mm, corrosion depth 1.25 mm, 2.5 mm, and 3.75 mm, respectively. The results show that eddy current field propagates around and to the depth after the direct section of the exciting current is cut off and the intensity center of eddy current field shifts gradually from the inner side of the tubing to the casing, which forms the basis of analyzing inspection mechanism. Corrosion at a particular depth is related to a particular optimum time slice of the induced voltage (namely with deepest concave) and a highest sensitivity is obtained at this slice. The time associated with this slice is in accordance with the time when the intensity center of eddy current reaches the corrosion. Corrosion at different depths has different voltage time slices starting to show signal of defect, which can be used to estimate the depth of the defect in order to judge the defect coming from tubing or casing. Furthermore, sinking degree of the time slice reflects the size of the defect. All machined defects can be recognized in the experiments and the optimum time slice appears at 0.01 s and 0.008 s after the excitation current is cut off for the tubing corrosion of 1.25 mm and 2.5 mm, respectively. The optimum time slice appears at the last moment of cut-off period, 0.625, for the casing corrosion. Experimental results agree well with the simulations and show the existence of the optimum correspondence between depth of corrosion and starting time of the defect signal of time slice, relations between sinking degree of the time slice, and corrosion size.

## 1. Introduction

In the process of oil exploitation, the integrity of oil casing is a guarantee of stable production. The tubing is inserted into the casing from the earth’s surface to the oil layer and is used as a pumping unit to transport oil to the surface. In the oil well, the casing plays a role in protecting the borehole, reinforcing the wellbore, and isolating the oil and gas layers. Due to the electrochemical corrosion, acid corrosion, crevice corrosion, fatigue corrosion, erosion corrosion and cavitation corrosion [1,2,3], as well as complex geological and engineering factors, there is always tubing and casing corrosion, deformation, cracking, and other damages affecting oil well safety and causing economic losses [1,2]. There are many methods for detecting the tubing. For casing, the inspection is taken mainly after taking out the tubing, which not only affects the production, but also prolongs the inspection cycle and increases the testing cost [4,5].

Pulsed eddy current (PEC) testing has become a new electromagnetic logging technology in recent years. Its feature is that the tubing and casing can be detected simultaneously by a probe which is placed in the tubing without taking out the casing. This method improves the inspection speed, saves the inspection cost, and facilitates the monitoring and survey of the oil well. The Russia All-Union Research Institute of Well Logging is leading in technology. In recent years, some oilfields in China have successively introduced electromagnetic logging tools such as EMDS-TM-42 and MID-K which are using pulse eddy current technology. Many Chinese technicians, such as Yancai Sun in Daqing Oilfield [6], Liangfeng Zhou [7] in Jiangsu Oilfield, Hongliang Liu et al. [8] in Tuha Oilfield, and Fengyang Xu in Zhonghai Oilfield Service Company [9], have applied them to detect some old oil wells, documenting their findings in several papers. However, their focus is mainly on the introductions of application but lack the specialized analysis of the inspection mechanism.

There is few basic research in this area. Damhuji Rifai et. al. studied the conventional eddy current testing method of pipeline [10,11]. Wei Zhang et. al. studied the pulsed far-field eddy current testing technique for ferromagnetic pipeline [12,13]. Yuewen Fu et. al. proposed a comparison standard for inspection sensitivity and compared the inspection characteristics of different probes [14,15]. Yu Song et. al. carried out numerical simulation of the time domain electromagnetic response of the longitudinal magnetic probe and the transverse magnetic probe in the device [16]. However, there is no further analysis and revealing of the dynamic changes of the eddy current field and its relationship with the inspection sensitivity. Products and research close to the tubing and casing pulse eddy current testing technology focus on using this technology to detect coated ferromagnetic pipes with wider application [17,18,19,20,21,22]. The results of these studies have proven that the pulse eddy current technology is effective for the inspection of metal defects in ferromagnetic components, which constitutes a reference for tubing and casing inspection. However, due to the probe placed outside the tube, the inspection arrangement and electromagnetic distribution are quite different. In order to facilitate the development of this technology, further research is needed.

The purpose of this paper is to conduct basic research on the inspection of tubing and casing to provide a mechanism and method for equipment development and application. For the corrosion inspection situation, the propagation of the eddy current field in the pipeline is numerically simulated, and the correlation between the propagation and the voltage time slice is studied. The sensitivity difference between different time slices is studied, and the inspection law is obtained.

This research is also applicable to the simultaneous inspection of the inner and outer layers of the double layer pipeline from inner layer. The double layer pipeline is sometimes used in other places than oil wells, such as submarine pipelines and pipelines crossing traffic lines.

## 2. Basic Principle of Pulse Eddy Current Detecting Tubing and Casing

**Figure 1:**
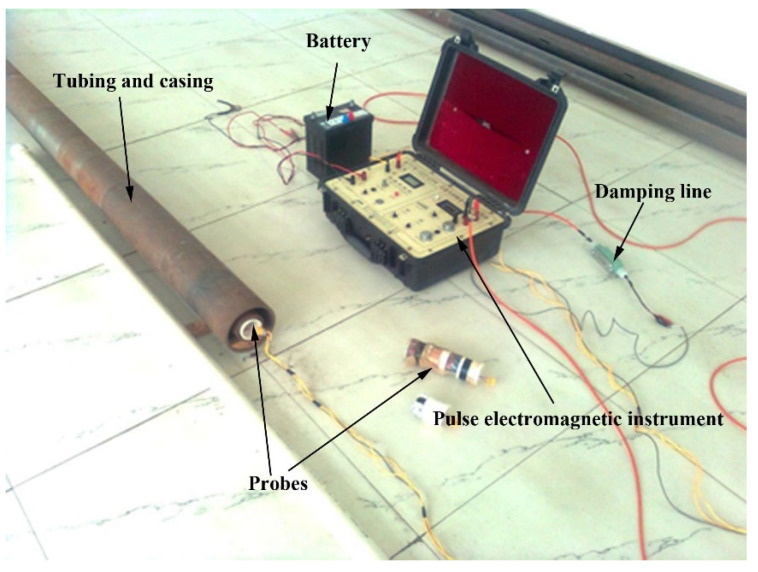
Experiment platform for tubing and casing inspection with PEC method.

**Figure 2:**
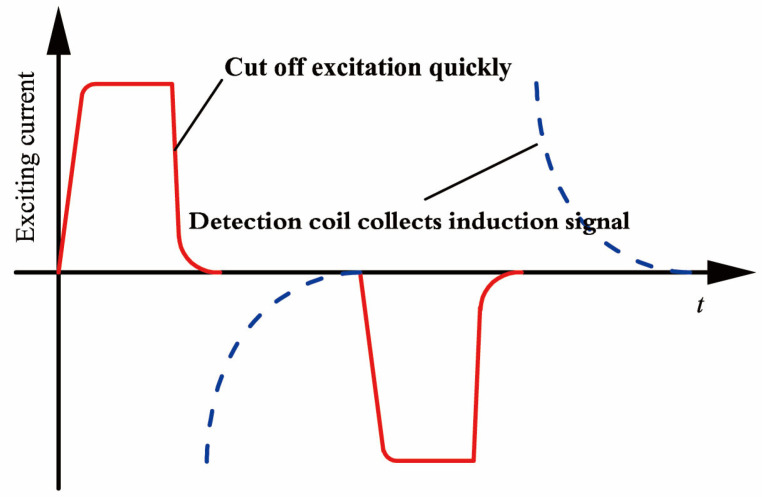
Diagram of pulsed eddy current excitation and reception signals.

Experiment platform for tubing and casing inspection with PEC method is shown in Figure 1. The platform consists of a pulse electromagnetic instrument, battery, damping line, testing sample, and the probe. The pulse electromagnetic instrument provides the pulse excitation current shown in Figure 2 and conducts data acquisition in the experiments. Damping line contains a resistor to adjust circuit damping characteristics of the receiving circuit. The testing sample contains tubing and casing, with tubing put inside the casing. The probe is placed inside the tubing.

The low frequency bipolar pulse current is exerted to the excitation coil by the pulse electromagnetic instrument to generate the excitation signal, as shown in Figure 2. During the period of DC section of the excitation, the casing will be in the steady primary magnetic field generated by the excitation coil. When the excitation signal is cut off quickly, the magnetic field nearby will change abruptly, which will induce pulsed current in the tubing and the casing. The induced current will generate a secondary magnetic field and the field will decay during off time of the excitation. Since the metal defect of the oil casing causes the secondary magnetic field to decay faster, the method of measuring the induced voltage drop curve of the receiving coil of the probe can be used to determine the corrosion and depth of the pipe.

## 3. Results

### 3.1. Experiment Apparatus

**Figure 3:**
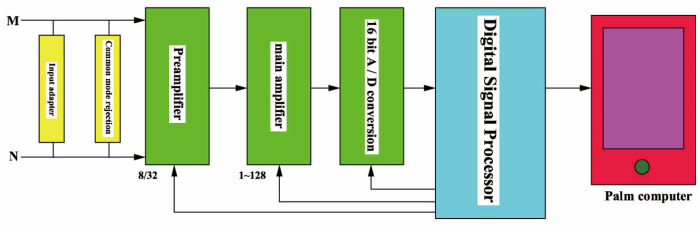
Principle block diagram of receiver.

The experimental equipment is a pulse electromagnetic instrument (containing transmitter, receiver, palm computer, etc.), which can provide a maximum peak current 10A, an equal width bipolar square wave pulse current with frequency from 1/16 Hz to 32 Hz. During the excitation off-period, the induced voltage signal on the receiving coil is collected, and the acquisition is 16-bit A/D, the highest sampling frequency is 1 MHz, and the two-stage amplification gain is up to 32 times ×128 times. Principle block diagram of the receiver is shown in Figure 3.

### 3.2. Test Specimens

**Figure 4:**
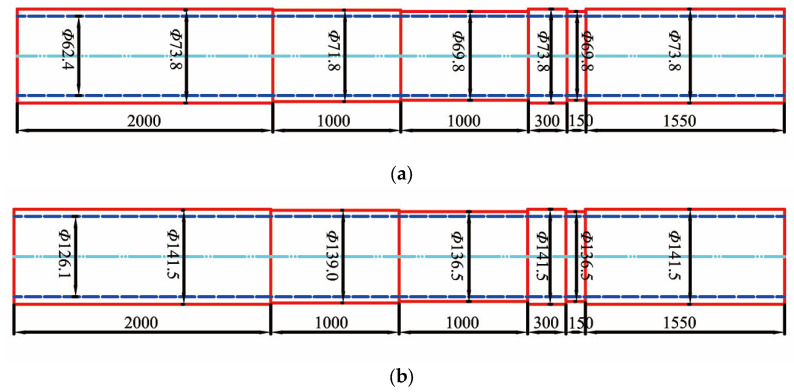
Specimens for tubing and casing inspection: (**a**) Corroded tubing; (**b**) Corroded casing.

The pipeline specimen is J55 special steel pipeline for an oil and water well, 4 in total. One inner tube (tubing) and one outer tube (casing) are intact, and the actual dimensions are *Ф*73.8 mm × 5.7 mm and *Ф*141.5 mm × 7.7 mm, and the length is 6 m. Another inner tube and outer tube were taken to make artificial corrosion defect specimens, as shown in Figure 4.

### 3.3. Probe Design

**Figure 5:**
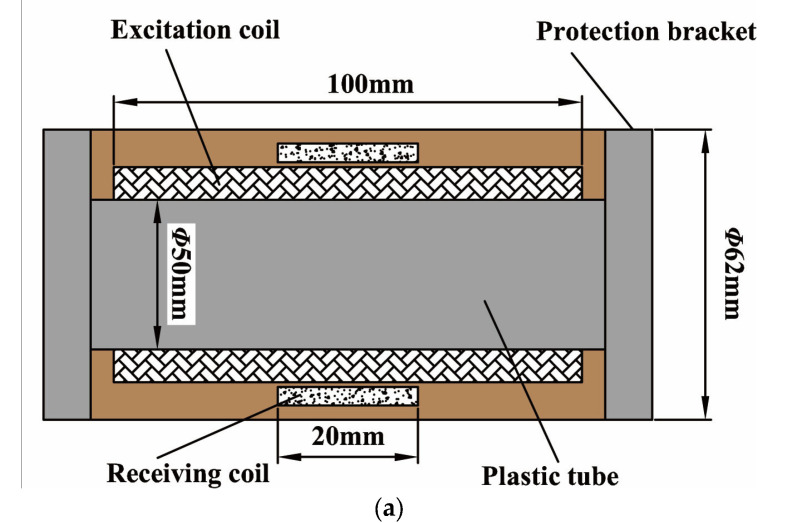

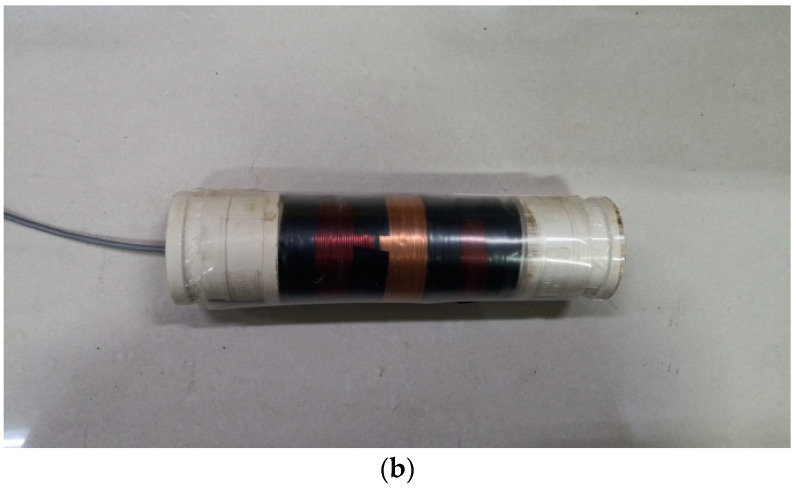
Specifications of the probe: (**a**) Probe; (**b**) Photo of the probe.

The probe consists of an excitation coil and a receiving coil which are coaxial and wound on a plastic tube. The excitation coil is an enameled wire with a wire diameter of 1 mm, and the receiving coil is an enameled wire with a wire diameter of 0.2 mm. The excitation coil is totally wound for 100 turns with two layers, and the reception is wound for 600 turns with 6 layers. Two protection brackets are made and fixed at two ends for protecting the probe. The probe size and photo are shown in Figure 5.

### 3.4. Processing Method of Inspection Data

During simulation or experimental inspection, the probe moves in the pipeline along the axial direction, and an inspection point is selected every certain distance for inspection. A total of *M* measuring points is set for the whole pipeline. The period of the induced voltage drop curve of the receiving coil of each measuring point is divided into 31 time windows according to the logarithmic distance. The signal spacing in the early stage is small, and the signal spacing in the later stage that changes slowly is large. Set *N* as the number of sampling windows for the induced voltage drop curve of each inspection point. Then the inspection data of the *i*-th point can be expressed as a vector [14]:

(1) $$V_{i}=\left[v_{i1}{,v}_{i2},\cdots {,v}_{\mathrm{iN}}\right]$$

The data of all *M* inspection points can be written into a matrix form:

(2) $$W=\left[\begin{array}{c} \begin{array}{c} V_{1}\\ V_{2} \end{array}\\ \begin{array}{c} \vdots \\ V_{M} \end{array} \end{array}\right]=\left[\begin{array}{cc} \begin{array}{cc} v_{11} & v_{12}\\ v_{21} & v_{22} \end{array} & \begin{array}{cc} \cdots & v_{1N}\\ \cdots & v_{2N} \end{array}\\ \begin{array}{cc} \vdots & \vdots \\ v_{M1} & v_{M2} \end{array} & \begin{array}{cc} \vdots & \vdots \\ \cdots & v_{\mathrm{MN}} \end{array} \end{array}\right]$$

where, $V_{i}$ is the induced voltage drop curve vector of the *i*-th point; $v_{iN}$ is the induced voltage of the *N*-th time window in the induced voltage drop curve of the *i*-th point; W is the matrix of *M* inspection points.

The row vector of the matrix ***W*** represents the induced voltage drop curve of a certain inspection point, and the column vector is the induced voltage at the same moment on the drop curve from the first inspection point to the *M*-th point. A curve is drawn taking the coordinates of the inspection point as the abscissa and the column vector as the ordinate. The curve is defined as the specimen inspection voltage time slice at the corresponding time of the column vector. If there is a defect at a certain inspection point, the induced voltage at this point on the voltage time slice of a column vector will be abnormal, and then the defect can be found. The induced voltage time slice curves will be exampled in the subsequent simulation and experimental data plots.

### 3.5. Evaluation Criteria for Inspection Sensitivity

Inspection sensitivity is defined to be a normalized difference of the voltage from the receiving coil for a certain corrosion defect relative to defect free area.

From Equation (2) time slices are obtained, *n*-th time slice can be expressed as a vector:

(3) $$U_{n}=\left[v_{1n}{,v}_{2n},\cdots {,v}_{\mathrm{Mn}}\right]$$

Set *i*-th inspection point in the pipeline is one with a corrosion defect and its receiving voltage at *n*-th time slice is *v*’*_in_*. If the *n*-th time slice voltage at other points in the pipeline without defect is *v_in_*, then the voltage difference between *v’_in_* and *v_in_* is Δ*v* = *v_in_*−*v’_in_*. Δ*v*/*v_in_* is the normalized voltage difference which is used as the inspection sensitivity for the defect at this time slice. The larger the Δ*v*/*v_in_*, the better the defect inspection effect of this time slice at this moment. Due to the noise disturbance, the signal is identified as a defect signal only when the Δ*v*/*v_in_* is larger than 5%.

## 4. Finite Element Simulation Analysis

### 4.1. Simulation Model Establishment

The specifications of the tubing and casing used in the simulation are the same as those of the actual pipeline shown in Figure 4, and the arrangement is shown in Figure 1 (tubing inside, casing outside). The corrosion depth of the tubing and casing is divided into three gradients: 1.25 mm, 2.5 mm, and 3.75 mm. The corrosion width was set to 100 mm in the simulation.

In the experiment, the pipeline was turned to different thicknesses in the circumferential direction to simulate corrosion at different depths, as shown in Figure 4. Under this kind of actual inspection object, the plane53 unit is selected in ANSYS during simulation, and then the model can be simplified to a two-dimensional symmetric model, which can greatly reduce the computational cost.

The actual tubing and casing surroundings are approximately processed. The gap between the longitudinal probe and the tubing, and the gap between the tubing and the casing, are set to air. The external of the casing is replaced by air. During calculating, the resistivity of the enameled wire is *ρ* = 1.72 × 10^−8^ Ω**•**m, the relative magnetic permeability is *μ_r_* = 1, and the relative permeability of air is *μ_r_* = 1. According to the J55 material parameters [23] of relevant data, the relative magnetic permeability of the tubing and casing is set to 164, and the resistivity is 2.5 × 10^−7^ Ω**•**m.

The loading of finite element model excitation is by coupling external circuit. Square pulses are applied to the excitation coil using CIRCU 124 unit. In the experiment, in order to eliminate the noise interference through symmetry, a bipolar pulse is used as the instrument. The bipolar pulse can better eliminate the background noise by averaging the signal collected during the positive pulse off-period and the signal collected during the negative pulse off-period. In the simulation calculation, the background noise interference is not considered, so the pulse is set to unipolar with frequency 8 Hz, duty ratio 1:1, and the amplitude of excitation current 1A.

### 4.2. Time Domain Dynamic Analysis of Eddy Current Field Distribution

The electromagnetic distribution of the pipeline during one inspection period is observed and analyzed by numerical simulation. The dynamic distribution of eddy current field has typical significance.

In the DC section excited, the magnetic field of the pipeline is approximately constant, and the eddy current field is approximately zero. After the excitation current is quickly cut off, the eddy current field will be induced in the pipeline. The distribution and variation of the eddy current field in the time domain will vary with the tubing and casing combination and defects.

**Figure 6:**
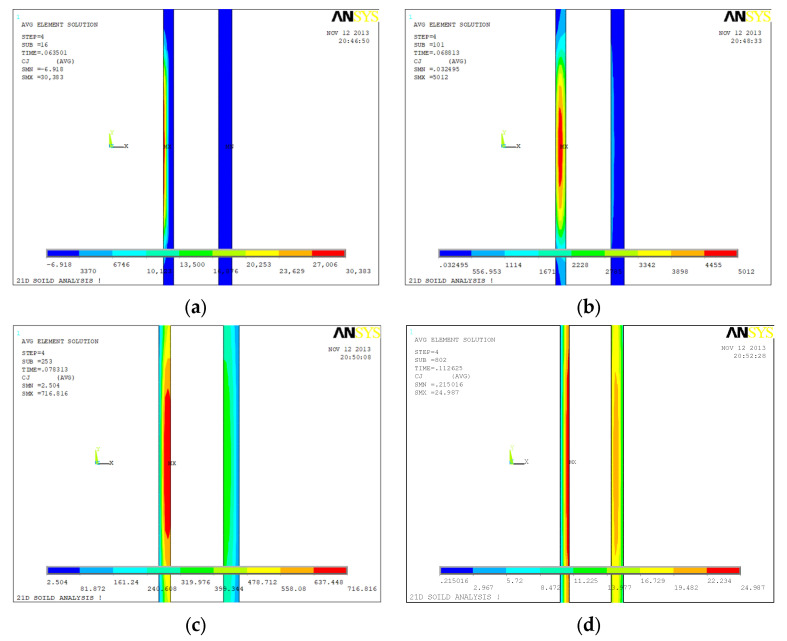
Eddy current distribution during power-off time in the inspection for undamaged casing plus undamaged tubing: (**a**) 0.0635 s (time slice No. 16); (**b**) 0.0688 s (time slice No. 22); (**c**) 0.0783 s (time slice No. 26); (**d**) 0.112 s (time slice No. 31).

**Figure 7:**
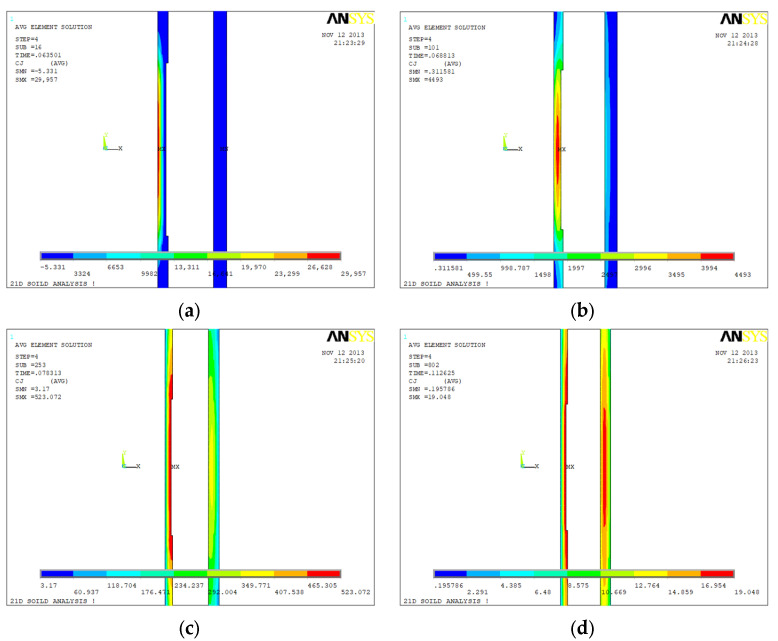
Eddy current distribution during power-off time in the inspection for undamaged casing plus corroded tubing: (**a**) 0.0635 s (time slices Nos. 16); (**b**) 0.0688 s (time slices Nos. 22); (**c**) 0.0783 s (time slices Nos. 26); (**d**) 0.112 s (time slices Nos. 31).

**Figure 8:**
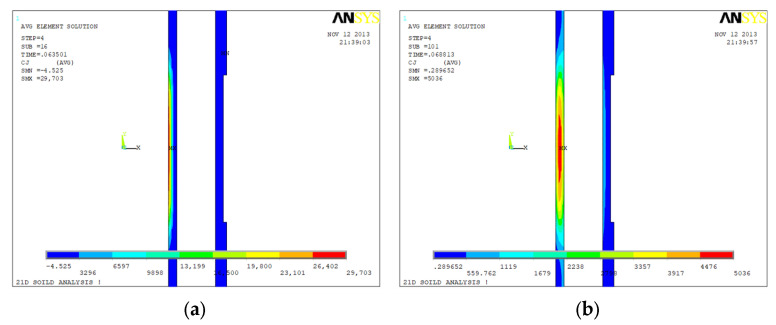

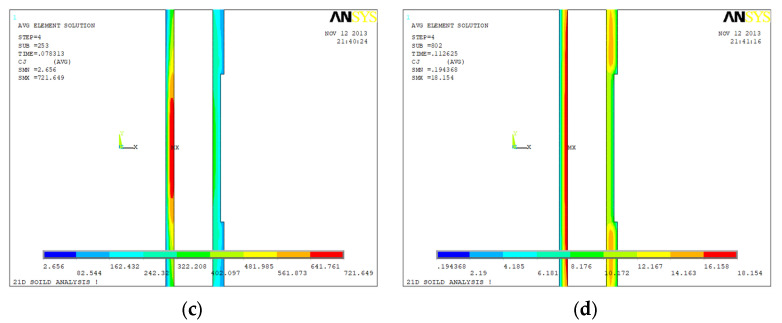
Eddy current distribution during power-off time in the inspection for corroded casing plus undamaged tubing: (**a**) 0.0635 s (time slices Nos. 16); (**b**) 0.0688 s (time slices Nos. 22); (**c**) 0.0783 s (time slices Nos. 26); (**d**) 0.112 s (time slices Nos. 31).

Figure 6, Figure 7 and Figure 8 show the eddy current distributions and their variations of the three tubing and casing combinations (internal and external all intact, internal corrosion external intact, internal intact external corrosion) during the period of the excitation current cutting off. Four moments were recorded: 0.635 s (time window No. 16), 0.688 s (time window No. 22), 0.783 s (time window No. 26), and 0.112 s (time window No. 31). The unit of eddy current density in the figure is A/m^2^.

Figure 6 shows the time domain distribution of the eddy current field of the intact tubing and casing combination after the current cut off. It can be observed that there are two features of the eddy current field after the excitation current cut off. First, the eddy current field is strong in the initiation, and its overall strength decreases rapidly over time. Second, the induced eddy current is not always strong on the surface and not always weak in the deep. During the signal receiving period of one pulse, except for the eddy current field expanding to the periphery, the intensity center of the eddy current field is gradually shifting from surface to depth, from the internal of the tubing to the casing. The second feature is the basis for the subsequent analysis of the inspection mechanism.

Figure 7 shows the time domain distribution of the eddy current field of the internal corrosion external intact combination after the current cut off. The corrosion is at the external of the tubing with a corrosion amount of 1.25 mm. The time domain distribution of the eddy current field of the intact tubing and casing combination after the current cut off was used as a reference. At 0.635 s, the intensity center of the eddy current field is at the internal of the tubing and the eddy current field distribution of the internal corrosion external intact combination is basically the same as that of the intact combination. At 0.688 s, the intensity center is close to the corrosion of the tubing and the eddy current field distribution is slightly different from that of the intact combination. At 0.783 s, the intensity center is at the corrosion of the tubing and the eddy current field distribution of these two combinations are quite different. At 0.112 s, there is still a large difference of the eddy current field distribution between the internal corrosion external intact combination and the intact combination.

Figure 8 shows the time domain distribution of the eddy current field of the internal intact external corrosion combination after the current cut off. The corrosion is at the external of the casing with a corrosion amount of 2.5 mm. At 0.635 s and 0.688 s, the eddy current field distribution of the internal intact external corrosion combination is basically the same as that of the intact combination. At 0.783 s, the intensity center of the eddy current field starts shifting to the casing and the eddy current field distribution is slightly different from that of the intact combination. At 0.112 s, the intensity center continues shifting to the corrosion, and the difference of the eddy current field distribution between the internal intact external corrosion combination and the intact combination is large.

Comparing the eddy current distribution of the defective tubing and casing with the intact ones based on Figure 6, Figure 7 and Figure 8, the eddy current distribution is similar at the initial moment of the excitation current cut off. When the intensity center of the eddy current field is at the defect, the difference of the distribution will be large.

The eddy current field distribution of the internal corrosion external intact with corrosion amount of 2.5 mm is similar to that of Figure 7, while the internal intact external corrosion with corrosion amount of 3.75 mm is similar to that of Figure 8. The distribution law is common.

### 4.3. Time Slice Analysis of the Receiving Coil Induced Voltage

**Figure 9:**
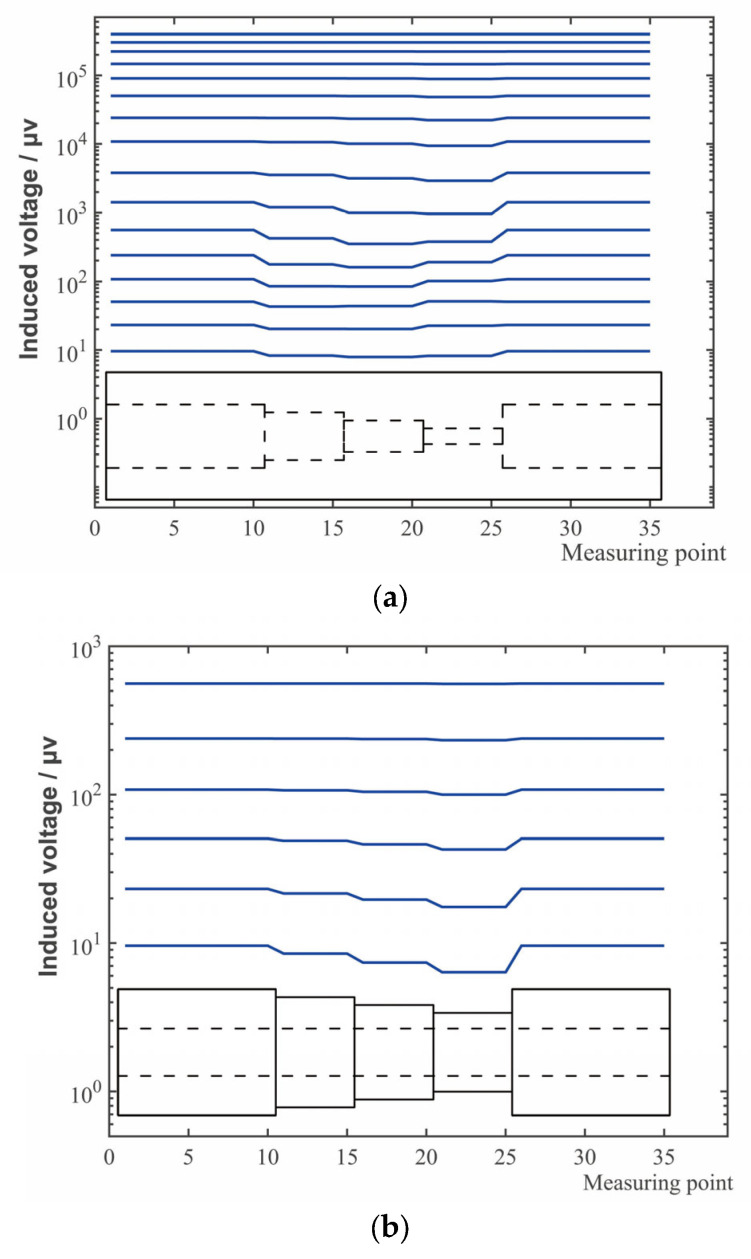
Time slices of voltage of receiving coil in the simulated inspection of tubing plus casing: (**a**) Corroded tubing plus undamaged casing; (**b**) Undamaged tubing plus corroded casing.

The induced voltages of the receiving coils of the internal corrosion external intact combination with corrosion depths of 1.25 mm, 2.5 mm, and 3.75 mm and the intact combination are extracted. The corrosions with three different depths are treated and placed in the same pipeline adjacently (easy to compare with the actual pipeline results). When the probe is located at the corrosion center, the induced voltage of the receiving coil is approximately taken as the induced voltage of each section of corrosion (the transitional effect at the step is simplified). According to Section 3.4, the induced voltages, which the 31 time windows obtained in the simulation corresponded to, are plotted as time slice, as shown in Figure 9a. In the same way, the time slice of the simulation induced voltages of the internal intact external corrosion combination is shown in Figure 9b.

It can be seen from Figure 9, that the concave surface in the time slice can reflect the presence of corrosions and corrosion amount. For internal and external defects, as well as different corrosion amounts, the time slice moment at which the defect signal starts to appear is different, and the inspection ability of each time slice to the defect is also different.

**Figure 10:**
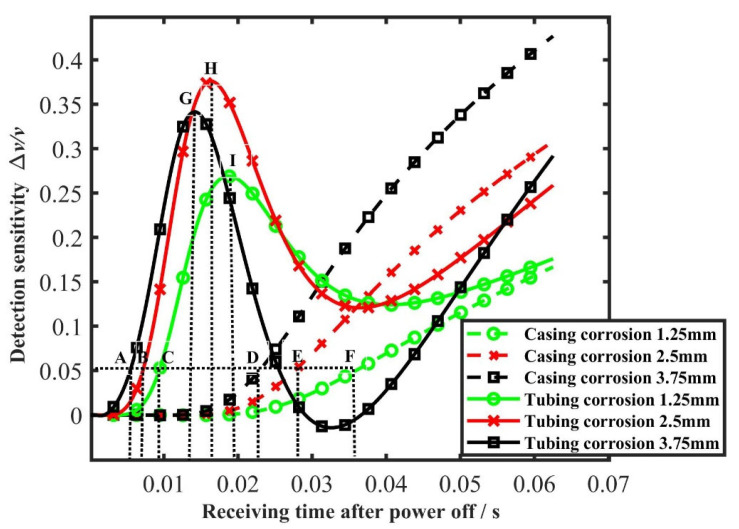
Sensitivity time slice curves for corrosion at different depths in the simulation.

In order to study the inspection sensitivity law of the time slice for the defect, the inspection sensitivity curve of the defect at each moment of the time slice is plotted, according to Section 3.5. Figure 10 shows the sensitivity curve of the induced voltage time slice at each moment under different corrosion depths of the internal corrosion external intact combination and internal intact external corrosion combination. When the inspection sensitivity *Δv/v* of the time slice reaches 5%, it is considered that the noise limit is exceeded, and a defect is determined to occur. The moment that the sensitivity peak corresponds to is the optimal inspection time slice moment. The moment that the normalized curve reaches 5% under several corrosion conditions can be obtained from Figure 10. The moments that the sensitivity reaches the peak are shown in Table 1. The absolute value in the table is the time after the current cut off plus the early-stage time 0.0625 s. The points in the table are labelled in Figure 10.

**Table 1:** 5% time and peak time of sensitivity time slice curves for different corrosions in the simulation.

Corrosion Depth	Tubing Corroding 1.25 mm	Tubing Corroding 2.5 mm	Tubing Corroding 3.75 mm	Casing Corroding 1.25 mm	Casing Corroding 2.5 mm	Casing Corroding 3.75 mm
Up to 5% moments (after power off) /s	0.0094 (point *C*)	0.0072 (point *B*)	0.0054 (point *A*)	0.0359 (point *F*)	0.0275 (point *E*)	0.0229 (point *D*)
Up to 5% moments (absolute value) /s	0.0719	0.0697	0.0679	0.0984	0.0900	0.0854
Peak time (after power off) /s	0.0196 (point *I*)	0.0164 (point *H*)	0.0142 (point *G*)	0.0625 (end)	0.0625 (end)	0.0625 (end)
Peak time (absolute value) /s	0.0821	0.0789	0.0767	0.1250	0.1250	0.1250

It can be observed from Figure 9 and Figure 10 and Table 1, that the moment the tubing corrosion starts to be shown is earlier than that of the casing corrosion. When the corrosions are all on the tubing, the moment that the corrosion starts to be shown is earlier as the corrosion depth is larger. For corrosion on the casing, when the corrosion is deep, the time for the defect to appear is earlier. The same is true of the corrosions on the casing.

For the defect of a certain corrosion depth on the tubing, there is an optimal inspection moment. The time slice at this moment has the best inspection sensitivity for the corrosion of this depth, and the sensitivity may change before and after this moment. The feature of this sensitivity variation causes, in the tubing inspection as shown in Figure 9a and for the time slices in later stage when the corrosion is large, the depression to be shallow instead. For casing, the sensitivity curve of time slice remains rising before the end of the receiving time, so the moment that the sensitivity reaches the maximum is the final moment. Therefore, for the inspection of tubing, the mid-term time slice should be selected to better reflect the corrosion degree. For casing, the inspection effect of later stage time slice is better.

Comparing the results of Figure 9 and Figure 10 and Table 1 with Figure 8, the moment that the center of eddy current intensity reaches the pipeline defect is consistent with the moment that the time slice reaches the optimum. When the center of eddy current intensity shifts to the corrosion defect, the proportion of the information in the total information is more prominent than that of other areas. The information difference caused by the defect is highlighted at this place. The time slice at this moment has the best inspection sensitivity for this defect. Therefore, it can be considered that the core of the inspection mechanism lies in the shifting of the center of the eddy current intensity.

It can also be seen that, the deeper the corrosion depth is, the deeper the time slice depression is. However, the relationship between the corrosion depth and the time slice depression will be different according to different time slices. A single time slice cannot provide accurate quantitative information of the defect.

## 5. Experiments and Analysis

### 5.1. Experiments

The devices shown in Figure 1 and the vertical probe shown in Figure 5 (the probe parameters are the same as that of the probe used in the simulation) are used to detect defective pieces and the intact piece shown in Figure 4. The amplitude of the pulse signal is set to 1A, which is the same as in the simulation. The fundamental frequency is 4 Hz (bipolar, and is unipolar 8 Hz in simulation). The spacing of adjacent inspection points (that is, the distance between two inspection piles) is 10 mm.

**Figure 11:**
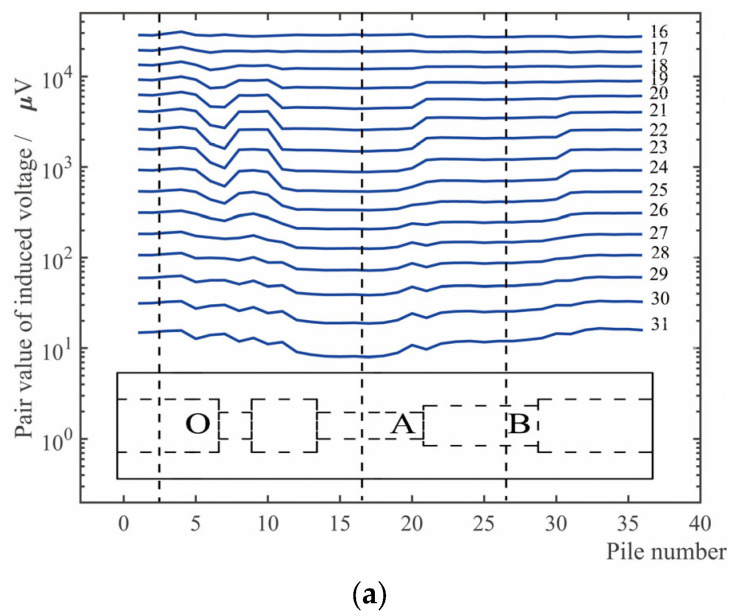

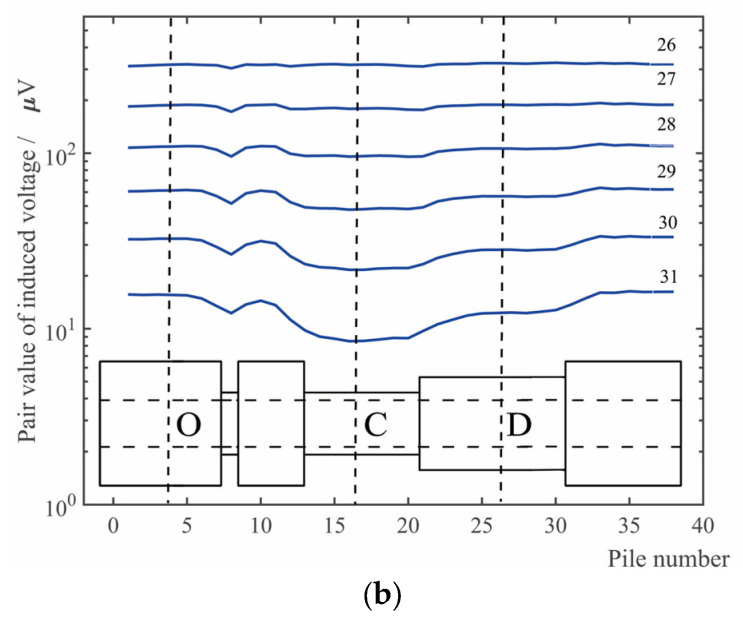
Time slices of voltage of receiving coil in the inspection of tubing plus casing: (**a**) Corroded tubing plus undamaged casing; (**b**) Undamaged tubing plus corroded casing.

Figure 11 shows the induced voltage time slice of the receiving coil of the internal corrosion external intact combination and the internal intact external corrosion combination. Several parts are marked in the figure: *O* represents the position that the internal and external are both without defects; *A* and *B* represents the positions of the corrosion with a large area and different depths in tubing; *C* and *D* represents the positions of the corrosion with a large area and different depths in casing.

**Figure 12:**
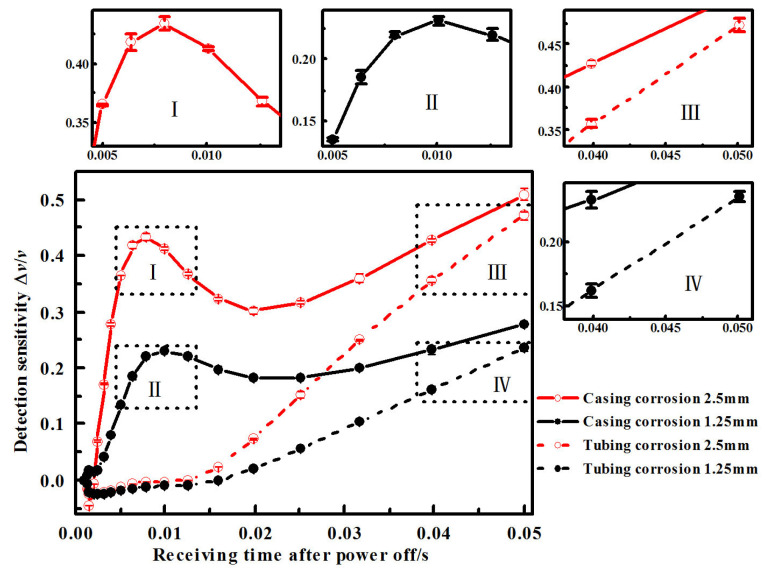
Sensitivity time slice curves for corrosion at different depth in the experiments.( The small graphs with Roman numerals are enlarged graphs of the corresponding part of the main graph).

Figure 12 shows the sensitivity curve of the induced voltage time slice under different corrosion depths of the undamaged casing plus corroded tubing combination, and the corroded casing plus undamaged tubing combination under three repeated experiments; the small images with Roman numerals are partially enlarged images. The receiving time is divided into 31 time windows to collect signals, so the moment of the first time window that exceeds 5% is used as the starting time for judging the appearance of the defect signal. The moments that the induced voltage sensitivity curve reaches 5% and peak, under various corrosions, can be obtained from Figure 12, as shown in Table 2. Each labelled point in the figure corresponds to the table.

The length of the probe used in the simulation and experiment is not greater than the width of the corrosion defect. For a defect whose width is smaller than the length of the probe, the defect can still be detected, but the inspection sensitivity will decrease. In some related work [14], a 300 mm-length probe was used to detect a pipeline corrosion whose width is 150 mm. Compared to a corrosion of the same depth but whose width is 1000 mm, the 150 mm width defect was detectable, but the inspection sensitivity was significantly decreased.

### 5.2. Analysis and Discussion

Comparing the simulation Figure 9 with the experimental Figure 11, it can be seen that the corrosion and corrosion degree of the pipeline can both be reflected by time slice of the simulation and the experiment in a certain period of time.

Comparing the experiments indicates there is an additional step with larger corrosion (corrosion amount is 3.75 mm, the wall thickness of tubing is 5.7 mm) in simulation. In the later stage time windows (31st window for example), a larger corrosion causes a shallower time slice depression instead. Combined with the inspection sensitivity curve of Figure 10, it can be seen that the corrosions with different depths correspond to different optimal inspection time slice, and the inspection sensitivity of partial time slices in the later stage is low for 3.75 mm corrosion. Therefore, the time slice depression induced by 3.75 mm corrosion is instead less than that of 2.5 mm corrosion. In the experiment, the large difference lead to a depression inversion that did not appear in the two corrosion steps, but the same law existed. Therefore, the time slices of the optimum inspection sensitivity corresponded to different depth corrosions are different, and an appropriate time slice should be selected for inspection. Under this inspection condition, the mid-term time windows should be utilized in tubing inspection, and the later stage time window should be utilized in casing inspection. A single time slice cannot reflect the size of the defects at different depths, and multiple time slices should be selected for defect quantification.

It can also be seen from the comparison of the simulation shown in Figure 10 and the experimental shown in Figure 12 that the overall trend of the inspection sensitivity variation of various types of corrosion is consistent in the simulation and experiment. The trend of the inspection sensitivity variation of tubing is rising, falling, and then rising, and that of casing is continuously rising. There is a certain complexity in the variation of inspection sensitivity. Therefore, an appropriate period should be selected when using time slice for inspection, and the time slice with weak inspection capability should be avoided. According to the basic laws of electromagnetic fields, the phenomenon of the tubing inspection sensitivity rising, falling, and then rising is related to the distribution of eddy current. Since the casing thickness is limited, the eddy current is limited by space. The later eddy current variation is affected by the reflection of electromagnetic field from the casing external, so the sensitivity will not vary monotonically all the time.

The moments that the sensitivity reaches 5% and peak in Table 1 and Table 2 was specifically compared. Considering that there will be differences between the starting moment in experimental data collection and that in simulation, the time interval difference between the two is compared in the following.

The interval between the moments that the sensitivity of tubing with 1.25 mm and 2.5 mm reaching 5%: simulation 0.0022 s, experiment 0.0015 s.

The interval between the moments that the sensitivity of tubing with 1.25 mm and 2.5 mm reaching peak: simulation 0.0032 s, experiment 0.0020 s.

The interval between the moments that the sensitivity of casing with 1.25 mm and 2.5 mm reaching 5%: simulation 0.0084 s, experiment 0.0050 s.

The interval between the moments that the sensitivity of casing with 1.25 mm and 2.5 mm reaching peak: simulation 0.0000 s, experiment 0.0000 s.

Although there are some differences in simulation and experiment due to the selection of parameters such as magnetic permeability, the order of moments is the same, and the intervals are roughly the same.

It can be seen from the simulation and experiment that, when the time slice method is used to detect tubing and casing, for different locations of corrosion, the time slice inspection sensitivities are different at different moments. The reason is that the intensity center of the eddy current field will shift from surface to depth over time. When the intensity center is at the defect, the inspection sensitivity is the highest. Since the time that the intensity center shifts to the corrosions with different depths is different, the moment of the optimum time slice is different.

The shifting of the intensity center of the eddy current field is the inspection mechanism core.

How to use multiple time slices to accurately quantify defects requires further research.

Based on the research results, test samples with different sizes of machined corrosion on them need to be made, with these samples materials and sizes to be the same as the inspected tubing and casing combination as far as possible before starting a practical inspection. Additionally, calibrations need to be carried out using these samples in order to determine whether a recognized corrosion comes from tubing or casing as well as corrosion size in practical inspections. In calibration, the time of the time slice at which a defect signal starts to appear is used to determine where the defect comes from, tubing or casing. Based on the defect location, optimum time slices are selected to calculate the size of the corrosion based on the sinking degree of the defect signal on these time slices. Results from different slices are averaged and the average residual thickness of the tubing or casing at a particular inspection position is given. Locations and sizes of the defects in a practical inspection can be given based on the data from the calibration.

## 6. Conclusions and Future Work

In this research, fundamental theory and methods of inspecting tubing are investigated along with casing string simultaneously with probe inside tubing using pulsed eddy current testing by numerical simulations and experiments.

The distribution and variation of eddy current field are given in the finite element simulation for the inspection of undamaged and corroded casing and tubing combinations. The results show that eddy current field propagates around and to the depth after the direct section of the exciting current is cut off and intensity center of the eddy current field shifts gradually from the inner side of the tubing to the casing.

Time slice method is used to recognize, locate, and quantify the defect. The simulation and experimental results show that corrosion at a particular depth is related to a particular optimum time slice of the induced voltage (namely with deepest concave) and a highest sensitivity is obtained at this slice. Additionally, the time associated with this slice is in accordance with the time when the intensity center of the eddy current reaches the corrosion. Corrosion at different depths has different voltage time slices starting to show signal of defect, which can be used to estimate the depth of the defect, and to judge the defect coming from tubing or casing. Furthermore, sinking degrees of the time slice reflects the size of the defect.

All machined defects, with tubing outer diameter 73.8 mm, wall thickness 5.7 mm, corrosion depth 1.25 mm, 2.5 mm, 3.75 mm, and casing outer diameter 141.5 mm, wall thickness 7.7 mm, corrosion depth 1.25 mm, 2.5 mm, 3.75 mm, respectively, can be recognized in the experiments, and the optimum time slice appears at 0.01 s and 0.008 s after the excitation current is cut off for the tubing corrosion of 1.25 mm and 2.5 mm, respectively. The optimum time slice appears at the last moment of cut-off period, 0.625 s for the casing corrosion.

Experimental results agree well with the simulations and show the existence of the optimum correspondence between depth of corrosion and starting time of the defect signal of time slice, and relations between sinking degree of the time slice and corrosion size.

Defect signal is affected by many factors such as corrosion depth and area and it is difficult to quantify the corrosion only by voltage difference from time slice, particularly for localized corrosion. More research needs to be done to analyze different contributions from different factors to better quantify the corrosion.

Oilfield water flooding scaling is a common phenomenon in the development process of many oilfields. Serious scaling may lead to the detection probe not be moved smoothly, so a special pig or scaling agent should be used to remove scaling. At the same time, the induced voltage value of the detection coil may be affected by the scaling, which makes it difficult to identify defects. Further research will be carried out at a later time.

## Figures and Tables

**Table 2:** 5% time and peak time of sensitivity time slice curves for different corrosions.

Corrosion Depth	Tubing Corroding 1.25 mm	Tubing Corroding 2.5 mm	Casing Corroding 1.25 mm	Casing Corroding 2.5 mm
Up to 5% moments (after power off) /s	0.0040 (point *N*)	0.0025 (point *M*)	0.0250 (point *P*)	0.0200 (point *O*)
Up to 5% moments (absolute value) /s	0.0665	0.0650	0.0875	0.0825
Peak time (after power off) /s	0.0100 s (point *R*)	0.0080 (point *Q*)	0.0625 (end)	0.0625 (end)
Peak time (absolute value) /s	0.0725	0.0705	0.1250	0.1250

## Data Availability

Not applicable.
